# OSAS Severity and Occlusal Parameters: A Prospective Study among Adult Subjects with Comorbidities

**DOI:** 10.3390/ijerph19095517

**Published:** 2022-05-01

**Authors:** Valeria Luzzi, Federica Altieri, Gabriele Di Carlo, Mariana Guaragna, Valentina Pirro, Beatrice Marasca, Luisa Cotticelli, Marta Mazur, Paola Di Giacomo, Carlo Di Paolo, Marco Brunori, Gabriele Piperno, Giuseppe Magliulo, Agnese Martini, Emma Pietrafesa, Antonella Polimeni

**Affiliations:** 1 Department of Oral and Maxillo Facial Sciences, ”Sapienza” University of Rome, 00161 Rome, Italy; valeria.luzzi@uniroma1.it (V.L.); federica.altieri@uniroma1.it (F.A.); mariana.guaragna@uniroma1.it (M.G.); valentina.pirro@uniroma1.it (V.P.); beatrice.marasca@uniroma1.it (B.M.); luisa.cotticelli@gmail.com (L.C.); marta.mazur@uniroma1.it (M.M.); p.digiacomo@uniroma1.it (P.D.G.); carlo.dipaolo@uniroma1.it (C.D.P.); marco.brunori@uniroma1.it (M.B.); gabriele.piperno@uniroma1.it (G.P.); antonella.polimeni@uniroma1.it (A.P.); 2 Department of Sensory Organs, “Sapienza” University of Rome, 00161 Rome, Italy; giuseppe.magliulo@uniroma1.it; 3 Department of Occupational and Environmental Medicine, Epidemiology and Hygiene, Italian Workers’ Compensation Authority (INAIL), 00143 Rome, Italy; a.martini@inail.it (A.M.); e.pietrafesa@inail.it (E.P.)

**Keywords:** breathing, sleep disorder, apnea, obstructive sleep, comorbidities, OSAS

## Abstract

Introduction: OSAS is an emerging public health problem. Early diagnosis in adults with comorbidities is the gold standard to avoid complications caused by a late diagnosis. The aim of the study, part of the SLeeP@SA project, was to identify within a population with dysmetabolic comorbidities the association of occlusal clinical signs, defined by orthodontic parameters, and of the anthropometric phenotype, with the severity of OSAS. Materials and Methods: A dedicated questionnaire containing questions regarding the presence of deep bite, augmented overjet, partial edentulism, and bruxism was completed by clinic staff. OSAS was evaluated using an unattended home PSG device, which recorded the AHI value. BMI and neck circumference were also measured. The Kolmogorov-Smirnov test was performed to evaluate the association of the AHI with occlusal clinical signs. The significance was set at *p* ≤ 0.05. The association of AHI with BMI and neck circumference was evaluated with the Pearson correlation coefficient. Results: In total, 199 subjects were evaluated. No statistically significant association between occlusal parameters and AHI was found, while the AHI showed a positive correlation with BMI and neck circumference. The neck circumference seemed to be a better clinical predictor for OSAS severity than BMI, especially for females. Conclusions: These results highlight how the orthodontic clinical data alone are not sufficient to establish an association between occlusal anomalies and OSAS severity, but further investigation involving a specialist orthodontic diagnosis is necessary.

## 1. Introduction

Obstructive sleep apnea syndrome (OSAS) is one of the most common sleep-disorder breathing conditions [1,2,3]. It is characterized by repetitive episodes of upper airway obstruction, causing cessation (apnea) of or reduction (hypopnea) in airflow during sleep, increasing daytime sleepiness, impaired cognitive function, and poor health status [4,5]. According to the Wisconsin Sleep Cohort study, the prevalence of OSAS was 24% in men and 9% in women aged 30–60 years [6].

OSAS patients are at increased risk of cardiovascular morbidity, hypertension, stroke, heart failure, diabetes, and mortality including sudden death [7,8]. One of the major issues when dealing with OSAS is the condition of underdiagnosis, which characterizes this group of pathologies. This is particularly relevant because OSAS is associated with sleepy driving and an increase in road accidents [1,9,10]. From this point of view, it is fundamental to develop efficient strategies to screen and prevent the comorbidities and the social impact caused by OSAS.

Therefore, it is important to identify risk factors for OSAS. The literature reports aging, male sex, large neck circumference, obesity, sleeping body position, hypertension, and lower oxygen saturation as risk factors in OSAS subjects [11,12,13,14,15,16]. Recently a large-scale survey, the HypnoLaus study, identified in OSAS subjects various associated comorbidities such as snoring, diabetes, hypertension, and metabolic syndrome [17]. This underlines the need to extend the screening of respiratory disorders during sleep in subjects exposed to OSAS risk factors.

Since 2018, the “SLeeP@SA” project has studied the prevalence of OSAS in the adult worker population with systemic diseases, namely patients at high risk to develop OSAS [18].

Craniofacial skeletal factors influence upper airway volume; therefore, the maxillary, mandibular, and hyoid bone position play a role in the pathogenesis of sleep apnea: subjects with OSAS typically have mandibular or bimaxillary retrognathism and a low-lying hyoid bone [19,20,21].

On the other hand, few studies describe the association between OSAS and occlusal parameters [22]. Still debated is also the association between sleep bruxism and sleep apnea. Currently there is no scientific evidence to support a conclusive relationship between sleep bruxism and OSAS [23,24]. According to a recent review, further studies are needed to investigate whether sleep bruxism shares a common pathogenesis with OSAS [25].

The primary goal of the study, part of the SLeeP@SA project, is to identify within a population with dysmetabolic comorbidities the association of occlusal clinical signs, defined by orthodontic parameters, and of the anthropometric phenotype, defined in terms of BMI and neck circumference, with the severity of OSA, measured by the apnea–hypopnea index (AHI).

## 2. Materials and Methods

This study was conducted at the Department of Oral and Maxillofacial Sciences of “Sapienza” University of Rome in the context of the BRIC INAIL project SLeeP@SA [18].

This project is an epidemiological survey whose goal is the early diagnostic screening of OSAS in the population of adult workers at greater risk of accidents. To this end, a three sections questionnaire was produced to be submitted to workers. The first and second sections of the questionnaire are self-reported and collect personal data and information about road accidents, occupational medicine, sleep quality through the Epworth Sleepiness Scale and Berlin Questionnaire, and ongoing pharmacological therapies. The third section is compiled by the clinical staff and contains information about clinical anamnestic data including height, weight, neck circumference, BMI, current health conditions, smoking habit, familiarity with pathologies, surgical operations, Friedman Tongue Position Classification, which describes the patency of the upper airways, dental examination, and polysomnography to measure AHI. In particular, the dedicated dental section contains questions regarding the presence of deep bite, augmented overjet, partial edentulism, corresponding to the absence of three or more teeth per arch, and bruxism.

All the patients completed the questionnaire and were monitored for two nights by an unattended home PSG device (SOMNOtouch™ RESP eco, SOMNOmedics GmbH, Randersacker, Germany). This equipment records the following parameters: nasal flow using cannula, thoracic, and abdominal movements, pulse oximetry, position sensor, and the AHI index. In particular, the AHI is computed through the variations in flow detected by the nasal cannula and by the thermistor, applied externally on the face at the level of the nose and mouth and matched with pulse–oximetry variations. The measure is considered valid if the PSG device was used at least six hours per night. The device is applied on the patient at the Department of Oral and Maxillofacial Sciences and returned after two days.

The study was reviewed and approved by the Ethics Committee of Policlinico “Umberto I” (No. 6131, 18 November 2020) and conducted in accordance with the Declaration of Helsinki. Patients were informed in detail of the purpose of the study, and written informed consent was obtained from all participants. All patients performing first access to the Department were subjected to anamnesis and clinical examination. Subjects between 18 and 65 years of age were asked to participate in the study if affected by one or more of the following OSAS risk factors, as confirmed by a medical examination: diabetes; obesity; metabolic syndrome; hypertension; heart disease; snoring.

### 2.1. Measurements of OSAS Severity

OSAS severity was measured by the AHI value, defined as the number of apnea and hypopnea episodes per hour averaged over the night, where an apnea is defined as a cessation of airflow through the mouth and nose for >10 s, and a hypopnea is defined as a reduction in airflow for >10 s associated with either oxygen desaturation of >3% or arousal. Individuals with AHI > 5 were diagnosed with OSAS. Following the AASM guidelines [26], OSAS severity was then classified as mild (5 < AHI ≤ 15), moderate (15 < AHI ≤ 30), and severe (AHI > 30).

### 2.2. Anthropometric Parameters

The Body Mass Index (BMI) was evaluated with the standard formula BMI = weight [kg]/height^2^ [m^2^].

Neck circumference was measured at the middle of the neck, between the mid-cervical spine and the superior line of the cricothyroid membrane, with the patient in a standing position.

### 2.3. Occlusal Parameters

The occlusal parameters detected during the intraoral clinical exam were: augmented overjet, defined as a distance between the lingual incisal edge of the most forwardly positioned maxillary incisor to the labial incisal edge of the most forwardly positioned mandibular incisor greater than 4 mm, and augmented overbite (deep bite), defined as a horizontal overlap of the upper incisors with the lower incisors greater than 4 mm [27]. Partial edentulism was clinically diagnosed while bruxism, defined as the involuntary grinding or clenching of teeth, was detected by abnormal tooth wear. 

### 2.4. Statistical Analysis

A descriptive analysis was performed on the data sample, including the distributions of AHI value and severity class. The association between OSAS severity and occlusal parameters was evaluated using the Kolmogorov-Smirnov test, with a significance set at *p* ≤ 0.05. The Pearson correlation coefficient of AHI vs. BMI, AHI vs. neck circumference, and BMI vs. neck circumference was measured, and the relative *p*-value was given, with a significance set at *p* ≤ 0.05. Data analysis was performed using the software SPSS version 25 (IBM corporation, Armonk, NY, USA) [28], and the ROOT framework version 6.24/04 [29].

## 3. Results

**Figure 1:**
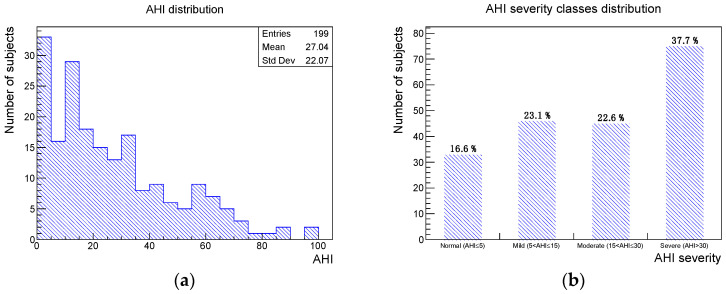
(**a**) AHI distribution and (**b**) AHI severity distribution for the study sample.

A total of 199 subjects, 128 males and 71 females, selected between January 2021 and February 2022 were considered in the current study. No data of interest were missing. The mean age was 53 years with a standard deviation of 11 years. The numbers and percentages of subjects of the sample presenting one of the four considered oral clinical signs were the following: deep bite 42 (21%), augmented overjet 27 (14%), partial edentulism 60 (30%), and bruxism 44 (22%). The AHI distribution, obtained from the PSG recording, and the associated severity class distribution are shown in Figure 1.

**Figure 2:**
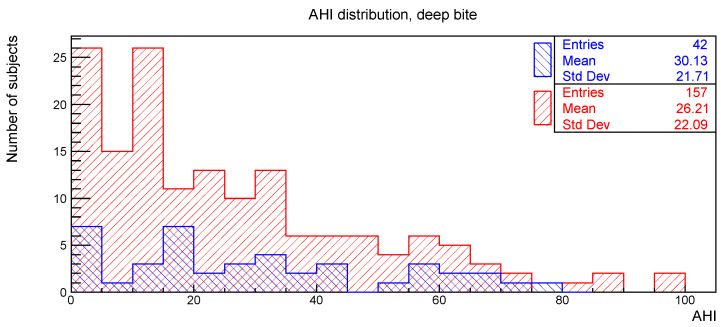
AHI distribution of subjects with (blue) and without (red) deep bite.

**Figure 3:**
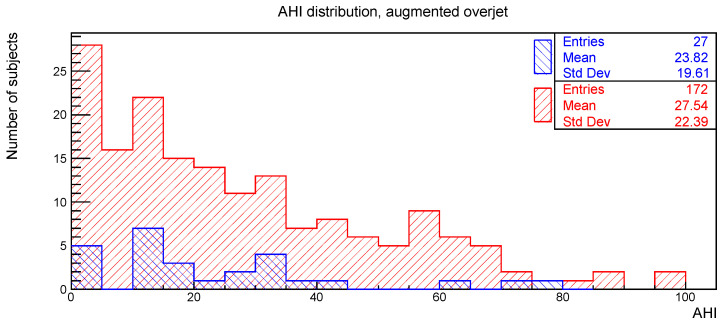
AHI distribution of subjects with (blue) and without (red) augmented overjet.

**Figure 4:**
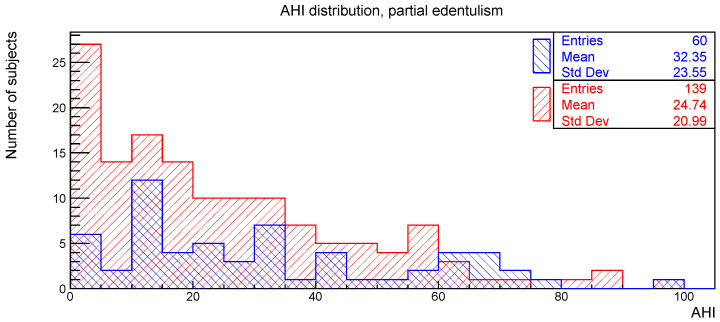
AHI distribution of subjects with (blue) and without (red) partial edentulism.

**Figure 5:**
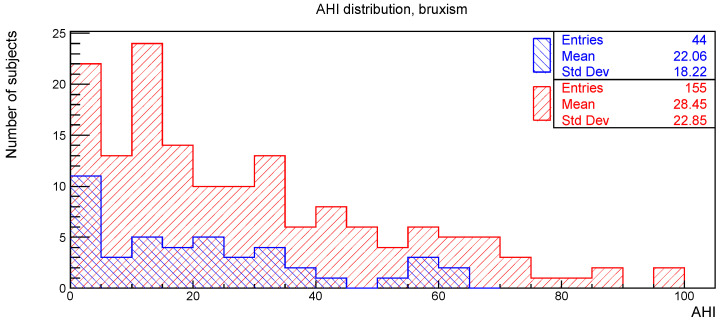
AHI distribution of subjects (blue) with and (red) without bruxism.

The distributions of the AHI value for subjects with or without the clinical signs of deep bite, augmented overjet, partial edentulism, and bruxism are reported in Figure 2, Figure 3, Figure 4 and Figure 5, respectively.

The relationships between AHI and deep bite, augmented overjet, partial edentulism, and bruxism were evaluated with the Kolmogorov-Smirnov test. The resulting *p*-values are shown in Table 1. No statistically significant difference was found.

Considering the differences in the body composition between males and females, the anthropometric analysis was split into these two groups. The Pearson correlation coefficients, the relative 95% confidence intervals, and the corresponding *p*-values for the correlation between AHI and BMI, AHI and neck circumference, and BMI and neck circumference for the male and female samples are shown in Table 2.

**Figure 6:**
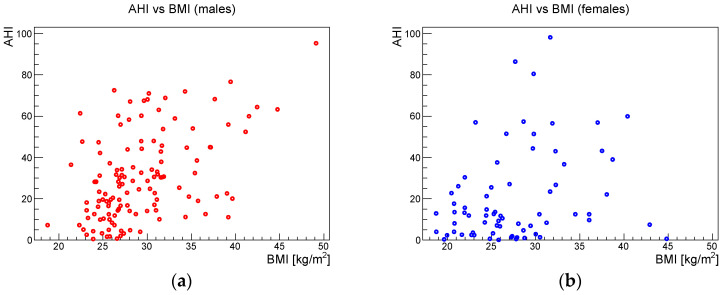
AHI vs. BMI for (**a**) males and (**b**) females.

**Figure 7:**
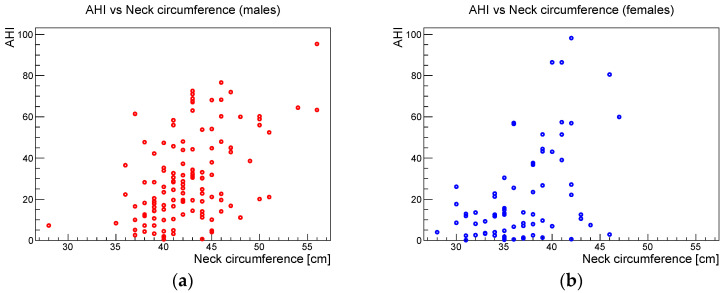
AHI vs. neck circumference for (**a**) males and (**b**) females.

**Figure 8:**
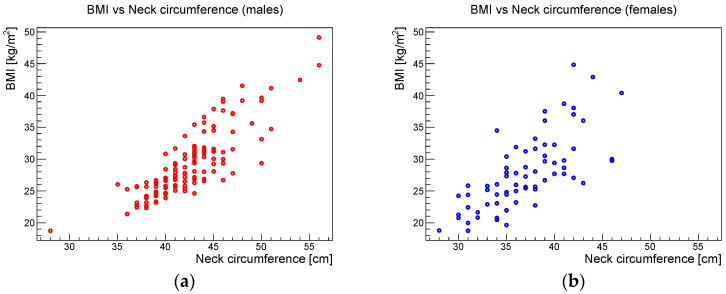
BMI vs. neck circumference for (**a**) males and (**b**) females.

Figure 6, Figure 7 and Figure 8 show the scatter plots of AHI vs. BMI, AHI vs. neck circumference, and BMI vs. neck circumference, respectively.

## 4. Discussion

The present prospective study evaluated the clinical oral signs among adult worker subjects with OSAS risk factors, afferent to the Department of Oral and Maxillofacial Sciences of “Sapienza” University of Rome. Many risk factors associated with the occurrence of OSAS are described in the literature [7,11,13,30]. Anatomical changes that contribute to oropharyngeal space reduction are among the most important of them [14,31,32]. Moreover, obese individuals with increased neck circumference, craniofacial alterations, and maxillo-mandibular deficiencies are at greater risk for OSAS [14,22,31]. In the current study, the association between OSAS severity, measured by means of the AHI, and the investigated occlusal clinical signs, namely augmented overbite (deep bite), augmented overjet, partial edentulism, and bruxism, was evaluated. No statistically significant relationships were found between the above clinical oral signs and the AHI. 

Miyao et al. investigated malocclusion of non-obese and obese patients with OSAS using cephalometric and dental model analyses [15]. The authors found a greater prevalence of deep overbite and severe overjet in patients with OSAS than in the general population and a significant correlation between AHI and overjet in non-obese patients. The same authors affirmed that severe overjet was closely linked to the severity of sleep-disordered breathing in non-obese patients suggesting that maxillofacial structures were a key factor for the OSAS severity [15].

Our lack of evidence of association of the occlusal parameters with AHI could be due to two factors: on the one hand, the associations were reviewed over the entire sample, and not stratified by risk factors. On the other hand, the survey of the occlusal parameters, for reasons of operational simplicity linked to the epidemiological nature of the project, was performed only through clinical observation and not through a quantitative assessment using cephalometric analysis and the study of plaster models. 

Our results are in agreement with Alqahtani et al. who used a simple method of dental examination and found no significant association between the different characteristics of molar, canine, and incisor occlusion and the severity of OSAS [22]. The authors underlined that the use of a simple method of dental examination, without the analysis of either cephalometric radiographs or study models, could be employed only as an initial assessment [22].

From this perspective, the role of the orthodontist is crucial. Indeed, in order to find a possible association between the occlusal parameters and the OSAS severity, a quantitative assessment method based on the measurement of the occlusion in all spatial planes and on cephalometric analysis in order to identify, e.g., micrognathism and retrognathism, is required.

Considering the partial edentulism in relation to AHI, no statistically significant association was found. Some authors investigated the relationship between tooth loss and signs and symptoms of obstructive sleep apnea and affirmed that tooth loss may be an independent risk factor for OSAS; in addition, complete tooth loss could favor the occurrence of upper airway obstruction during sleep [24,25]. 

Since the anatomical changes caused by the loss of posterior teeth consist in loss of the vertical dimension of occlusion, in the reduction in the lower face height, and in the rotation of the mandible, it is necessary to establish the type of edentulism that leads to the occlusion disharmony that favors the occurrence of OSAS [24]. In the present study, the type of data collection relating to edentulism did not provide a detailed analysis of the distribution of missing teeth, thus making it impossible to find any associations with OSAS.

Regarding the relationship between bruxism and AHI, in agreement with previous literature, in the present study no statistically significant association was found [23]. 

Concerning the anthropometric parameters analyzed in this study, a highly significant correlation was found between BMI and neck circumference without significant gender differences. Beyond this expected phenotypic association, the study showed that BMI and neck circumference showed a statistically significant association with AHI in both males and females. These results confirmed the data reported in the literature, which indicate that obesity, i.e., an increased BMI, is one of the main risk factors for OSAS in adulthood [15]. The study also showed that neck circumference had a stronger statistical association with AHI than BMI.

Our results should be interpreted, according to some limitations. In particular, as previously stated, the definition of oral signs was purely clinical, while an extensive differentiation of these signs could have been obtained by means of a complete cephalometric analysis. Nevertheless, due to the epidemiological approach of the project, radiographical assessments were not performed both for operative and ethical reasons. Another limitation of this study was related to the sample size; the relatively low prevalence of oral signs within the sample reduced the sensitivity of the statistical analysis. As the SLeeP@SA project is still ongoing, the final larger data sample will improve our sensitivity. Finally, it must be noted that the results obtained with the unattended home PSG device we used are slightly different from those of a full polysomnography, where arousals are detected via EEG. Nevertheless, the home PSG included the finger plethysmography to indirectly detect arousal in a way, which can be considered equivalent to the recording of the sympathetic hyper tone.

## 5. Conclusions

OSAS is a serious health problem that has important impacts on the life quality and expectancy of affected individuals. Based on the results of the present study, the clinical investigation of augmented overbite, augmented overjet, partial edentulism, and bruxism did not show a significant association with AHI severity among adult patients with comorbidities. These results need to be confirmed by studies with the aim to evaluate the impact of malocclusion severity on OSAS. The neck circumference can be a good easily measurable clinical predictor associated with OSAS severity.

## Figures and Tables

**Table 1:** Number of subjects, mean value, and standard deviation for the AHI distributions considered in Figure 2, Figure 3, Figure 4 and Figure 5, together with the *p*-values obtained from the Kolmogorov-Smirnov test between AHI and the corresponding clinical oral sign.

AHI vs.	With			Without			*p*-Value
	n. of Sub.	AHI Mean	AHI Std. Dev.	n. of Sub.	AHI Mean	AHI Std. Dev.	
Deep bite	42	30.1	21.7	157	26.2	22.1	0.28
Augmented overjet	27	23.8	19.6	172	27.5	22.4	0.67
Partial edentulism	60	32.3	23.6	139	24.7	21.0	0.22
Bruxism	44	22.1	18.2	155	28.4	22.8	0.28

**Table 2:** Pearson correlation coefficient with the 95% confidence interval indicated in parenthesis and the relative *p*-value for the parameters given in the left column, for males and females.

	Males		Females	
	Corr. Coeff.	*p*-Value	Corr. Coeff.	*p*-Value
AHI vs. BMI	0.48 [0.33, 0.60]	9.0 × 10^−9^	0.26 [0.03, 0.47]	0.025
AHI vs. Neck circ.	0.50 [0.36, 0.62]	2.1 × 10^−9^	0.47 [0.27, 0.63]	2.8 × 10^−5^
BMI vs. Neck circ.	0.84 [0.78, 0.88]	<10^−12^	0.72 [0.58, 0.82]	1.0 × 10^−12^

## Data Availability

Data available on request due to restrictions. The data presented in this study are available on request from the corresponding author.
